# Quetiapine Attenuates the Neuroinflammation and Executive Function Deficit in Streptozotocin-Induced Diabetic Mice

**DOI:** 10.1155/2019/1236082

**Published:** 2019-01-17

**Authors:** Kexin Wang, Feng Song, Hongxing Wang, Jun-hui Wang, Yu Sun

**Affiliations:** ^1^Department of General Surgery, Qilu Hospital of Shandong University, Jinan, Shandong, China; ^2^Department of Orthopedics, Qingdao University Affiliated Qingdao Municipal Hospital, Qingdao, Shandong, China; ^3^Department of Neurology, Xuanwu Hospital, Capital Medical University, Beijing, China; ^4^University of Toronto, Department of Physiology, Toronto, Ontario, Canada; ^5^Department of Endocrinology, Qilu Hospital of Shandong University, Jinan, Shandong, China

## Abstract

Diabetic patients are at increased risk for developing memory and cognitive deficit. Prior studies indicate that neuroinflammation might be one important underlying mechanism responsible for this deficit. Quetiapine (QTP) reportedly exerts a significant neuroprotective effect in animal and human studies. Here, we investigated whether QTP could prevent memory deterioration and cognitive impairment in a streptozotocin- (STZ-) induced diabetic mouse model. In this study, we found that STZ significantly compromised the behavioral performance of mice in a puzzle box test, but administering QTP effectively attenuated this behavioral deficit. Moreover, our results showed that QTP could significantly inhibit the activation of astrocytes and microglia in these diabetic mice and reduce the generation and release of two cytokines, tumor necrosis factor-*α* (TNF-*α*) and monocyte chemoattractant protein-1 (MCP-1). Meanwhile, QTP also prevented the protein loss of the synaptic protein synaptophysin (SYP) and myelin basic protein (MBP). Here, our results indicate that QTP could inhibit neuroinflammatory response from glial cells and block the injury of released cytokines to neurons and oligodendrocytes in diabetic mice (DM). These beneficial effects could protect diabetic mice from the memory and cognitive deficit. QTP may be a potential treatment compound to handle the memory and cognitive dysfunction in diabetic patients.

## 1. Introduction

Diabetes mellitus (DM) is a chronic metabolic disorder characterized by abnormally high levels of glucose in the blood. Over 220 million people around the world have diabetes, a statistic that represents 8.3% of the population. The number of people is estimated to double by the year 2030 [1]. The deleterious effects of DM on the central nervous systems (CNS) are attracting attention recently. Both type-1 and type-2 DM patients showed reduced performances on some types of memory and cognitive function [2]. Because of the costs of health care and long-term care individuals, especially elderly patients with DM and other dementia, the economic burden is very substantial. Although DM is associated with vascular risk factors of memory and cognitive deterioration, diabetes itself has been considered as an independent predisposing risk factor for the brain function deficit [3]. But overall effective therapeutic strategies for the memory and cognitive dysfunction in DM patients are not available yet due to lack of understanding of their pathogenesis. Therefore, exploring the possible underlying mechanism and potential compound to handle this disorder is imperative.

Diabetes and psychiatric disorders share a bilateral association, both influencing each other in many ways [4]. QTP has been used in the treatment of psychosis and some mood disorders [5]. In elderly patients, QTP exerts a relatively safe profile on treatment of agitation of patients with dementia [6]. Importantly, QTP has been shown to be a neuroprotectant [7]. >But no effort has been made to investigate the effect of QTP on the memory and cognitive dysfunction in DM patients.

Here, we hypothesized that QTP might be a potential candidate compound for the treatment of memory and cognitive deficit in DM patients. We tested the hypothesis in a STZ-induced DM mouse model. We found that QTP treatment could significantly protect the DM mice from memory and cognitive deficit. Furthermore, we found that QTP might inhibit the activation of glial cells and reduce the neuroinflammation in the brains of these DM mice, leading to the prevention of synaptic and myelin protein loss.

## 2. Materials and Methods

### 2.1. Animals and Drugs

8-week-old male C57BL/6J mice were group housed and maintained on a 12-hour light : 12-hour dark cycle with food and water for a 1-week acclimation period. All mice were treated according to the guidelines established by the Chinese Council on Animal Care, and all procedures were approved by the Animal Care Committee of the Qingdao University Affiliated Qingdao Municipal Hospital, China, and Qilu Hospital of Shandong University, China.

Mice were divided into 4 groups: control (*n* = 8), control plus QTP (5 mg/kg/day; *n* = 8), STZ (150 mg/kg; *n* = 8), and STZ plus QTP (5 mg/kg/day; *n* = 8). STZ and QTP were purchased from Sigma-Aldrich (MO, USA). STZ was dissolved in distilled 0.1 mmol/l sodium citrate buffer (pH 4.5), and the experimental dose of QTP was prepared in distilled drinking water (2 mg/100 ml water) as previously reported [7]. Single-dose intraperitoneal injection of STZ was administrated to induce a diabetic mouse model. QTP was given to mice in drink water 1 week before the single-dose STZ injection and lasted for 5 weeks. Behavioral tests were performed on the last week with QTP treatment. Mice were then sacrificed to collect the brain hippocampal tissues for western blot and biochemistry assays immediately after the behavioral test was done.

### 2.2. Puzzle Box Test

Executive function was assessed using the puzzle box assay, as reported previously [8, 9]. The apparatus is a PLEXIGLAS white box, consisting of an illuminated start box (58 × 28 × 27.5 cm^3^) and a dark, enclosed goal box (14 × 28 × 27.5 cm^3^). Mice were positioned in the open box, and then the latency that mice enter the close box was recorded. Mice underwent a total of nine trials (T1–T9) during 3 consecutive days with 3 trials per day: Day 1 (trials 1-3), Day 2 (trials 4-6), and Day 3 (trials 7-9). First, mice had to use an open doorway (T1) to enter the goal box. Then, the doorway was blocked, and mice had to pass through an open underpass to reach the goal box (T2). This underpass challenge was measured in T3 again, which allowed us to assess short-term memory and learning performance of these mice. On the second day of testing, T3 was assessed again to measure long-term memory and learning of the same task which corresponded to T4. On T5, the underpass was blocked with bedding and mice had to burrow through the bedding to enter the goal box. This challenge of burrowing was repeated in T6 (second day) and T7 (third day) to assess short- and long-term memory, respectively. On the third day, a cardboard plug was used to block the underpass (T8 and T9), and mice had to move the plug out of the underpass before they could enter the dark box. A 2-minute interval was set between trials in each day. During each trial, a maximum time of 5 minutes was given to each mouse to reach the goal box.

### 2.3. Collection of Cerebrospinal Fluid (CSF) Samples

CSF was collected from the cisterna magna, as described by a previous report with minor modifications [10]. Briefly, a sagittal incision was made inferior to the occiput after anesthesia with isoflurane, and the subcutaneous tissue and muscles in the surrounding area were removed. After exposing the meninges, a glass capillary tube was used to penetrate the meninges to collect 3-4 *μ*l CSF.

### 2.4. Western Blot Analysis

The extracted proteins from the hippocampus were separated by electrophoresis with the 12% sodium dodecyl sulfate polyacrylamide gel electrophoresis (SDS-PAGE) and then transferred onto nitrocellulose membranes. They were then electrophoretically transferred onto nitrocellulose membranes. The membranes were blocked with 5% (*w*/*v*) nonfat dried milk in TBST buffer and were probed with polyclonal rabbit antibody to glial fibrillary acidic protein (GFAP) (Millipore Corporation, MA, USA), CD11b (Abcam, UK), synaptophysin (SYP, Abcam, UK), and myelin basic protein (MBP, Abcam, UK) in TBST milk overnight at 4°C. The membranes were also probed with mouse monoclonal antibody to GAPDH (Abcam, UK) or *β*-actin (Abcam, UK) as a loading control (Santa Cruz Biotechnology, CA, USA). After incubation with the secondary antibodies for 2 hours at room temperature, antigens were revealed by a chemiluminescent reaction (Amersham Biosciences, NJ, USA). Quantitative results were expressed as a ratio of target protein to GAPDH.

### 2.5. Enzyme-Linked Immunosorbent Assay (ELISA)

The concentrations of MCP-1 and TNF-*α* in hippocampal tissue and CSF were assessed with ELISA kits (eBioscience, Thermo Fisher Scientific), following the manufacturer's protocol. Each sample of brain tissues was assayed in duplicate at appropriate dilutions so that relative luminescent units fell within the linear range of standard curves. The values of MCP-1 and TNF-*α* from each well was normalized and expressed as a ratio of the total loading protein in brain tissue. 2 *μ*l CSF was assayed in duplicate at appropriate dilutions so that relative luminescent units fell within the linear range of standard curves. The absorbance of each sample was measured at 450 nm by using a microplate reader (Synergy Mx, BioTek, Winooski, VT).

### 2.6. Statistical Analysis

All of the results are expressed as the mean ± SEM. The significance of differences was determined by one-way ANOVA, followed by the Bonferroni post hoc test for multiple comparisons. A *p* value of less than 0.05 was regarded as statistically significant.

## 3. Results

### 3.1. QTP Attenuated Memory and Executive Function Deficit in DM Mice in a Puzzle Box Test

**Figure 1:**
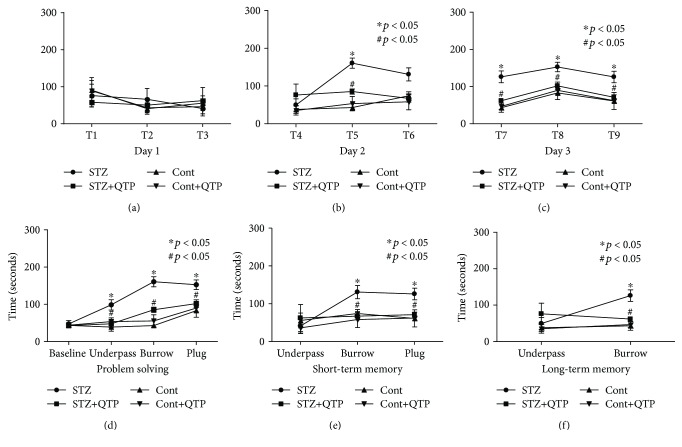
QTP attenuated memory and executive function deficits of DM mice in a puzzle box test. (a) Latencies of mice in each group to complete the task in T1-T3. (b) Latencies of mice in each group to complete the task in T4-T6. (c) Latencies of mice in each group to complete the task in T7-T9. (d) Latencies of mice in each group to solve a new problem during the 3-day test (T1, T2, T5, and T8). (e) Latencies of mice in each group to solve a repeat new problem after 3-minute intervals during the 3-day test (short-term memory) (T3, T6, and T9). (f) Latencies of mice in each group to solve a repeat new problem after 24-hour intervals during the 3-day test (long-term memory) (T4 and T7). All data were expressed as means ± SEM. ^∗^*p* < 0.05 STZ vs. Cont; ^#^*p* < 0.05 STZ+QTP vs. STZ, *n* = 8.

The puzzle box test assessed the latencies of mice to move from a bright box to an enclosed dark box. The results of the 9 trials over 3 consecutive days were analyzed with one ANOVA analysis (Figure 1). On the first day of testing, all mice spent almost an identical amount of time to enter the dark box in the first 3 trials (T1-T3) (Figure 1(a)). On the second day, DM mice had increased latencies to enter the goal box compared to mice in the control group during T5 when the underpass was filled with bedding (burrowing puzzle). In addition, QTP treatment was observed to decrease the latencies (Figure 1(b)) (*F*_(1, 28)_ = 40.14, STZ vs. Cont, ^∗^*p* < 0.001; *F*_(1, 28)_ = 7.517, STZ+QTP vs. STZ, ^#^*p* = 0.017; two-way ANOVA analysis). Significant difference was also observed on T7 (burrow puzzle) (*F*_(1, 28)_ = 19.69, STZ vs. Cont, ^∗^*p* = 0.001; *F*_(1, 28)_ = 7.479, STZ+QTP vs. STZ, ^#^*p* = 0.0107; two-way ANOVA analysis), T8 (plug puzzle) (*F*_(3, 28)_ = 5.81; STZ vs. Cont, ^∗^*p* < 0.05; STZ+QTP vs. STZ, ^#^*p* < 0.05; one-way ANOVA followed by Bonferroni post hoc analysis), and T9 (plug puzzle) (*F*_(3, 28)_ = 5.56; STZ vs. Cont, ^∗^*p* < 0.05; STZ+QTP vs. STZ, ^#^*p* < 0.05; one-way ANOVA followed by Bonferroni post hoc analysis) of the third day in DM mice compared to control mice, but this effect was reversed by the QTP treatment (Figure 1(c)). Next, we evaluated other parameters by analyzing the same data among these four groups: problem solving (T1, T2, T5, and T8, when mice encountered a new puzzle for the first time), short-term memory (T3, T6, and T9, when mice encountered the same puzzle twice in a time period of 5 minutes), and long-term memory (T4 and T7, when mice encountered the same puzzle twice in a period of 24 hours). The capacity of problem solving was measured with the latency of entering the enclosed box when mice were confronted with the series of challenges for the first time in each day. As shown in Figure 1(d), there was a statistically significant difference between control mice and DM mice on the latency of the underpass task (*F*_(3, 28)_ = 5.65; STZ vs. Cont, ^∗^*p* < 0.05; STZ+QTP vs. STZ, ^#^*p* < 0.05; one-way ANOVA followed by Bonferroni post hoc analysis), burrow task (*F*_(1, 28)_ = 41.57, STZ vs. Cont, ^∗^*p* = 0.001; *F*_(1, 28)_ = 7.149, STZ+QTP vs. STZ, ^#^*p* = 0.0106; two-way ANOVA analysis), and plug task (*F*_(3, 28)_ = 5.81; STZ vs. Cont, ^∗^*p* < 0.05; STZ+QTP vs. STZ, ^#^*p* < 0.05; one-way ANOVA followed by Bonferroni post hoc analysis). However, QTP treatment could effectively decrease the latency of DM mice. In the short-term memory test, there was no significant difference between all the groups in the underpass task (Figure 1(e)). However, DM mice spent a longer time to enter the goal box in the burrow (*F*_(1, 28)_ = 4.39, STZ vs. Cont, ^∗^*p* = 0.0479; *F*_(1, 28)_ = 6.486, STZ+QTP vs. STZ, ^#^*p* = 0.0184; two-way ANOVA analysis) and the plug (*F*_(3, 28)_ = 5.56; STZ vs. Cont, ^∗^*p* < 0.05; STZ+QTP vs. STZ, ^#^*p* < 0.05; one-way ANOVA followed by Bonferroni post hoc analysis) task after a short-term interval. Interestingly, QTP could significantly decrease the latency of DM mice in these two tasks. In the long-term memory test, there was no significant difference between all the groups in the underpass task as well (Figure 1(f)). However, DM mice spent a longer period of time to enter the goal box in the burrow (*F*_(1, 28)_ = 19.68, STZ vs. Cont, ^∗^*p* = 0.001; *F*_(1, 28)_ = 7.479, STZ+QTP vs. STZ, ^#^*p* = 0.0107; two-way ANOVA analysis) task after a long-term interval (24 hours). QTP could significantly decrease the latency of DM mice in this task. These results imply that these mice experienced executive and memory function impairment. In addition, it was observed that QTP treatment prevented both the executive and memory deterioration in these DM mice.

### 3.2. QTP Inhibited GFAP and CD11b Protein Expression Level in the Hippocampal Tissues of DM Mice

**Figure 2:**
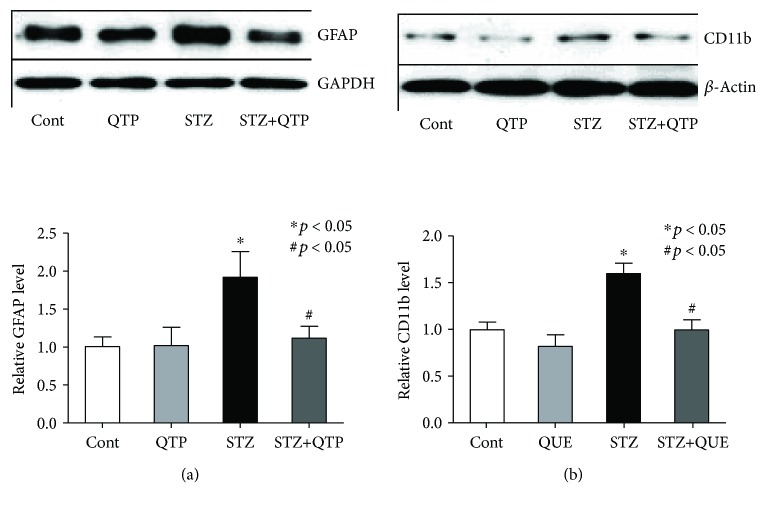
QTP inhibited GFAP and CD11b protein expression level in the brain hippocampal tissues of DM mice. (a) Representative bar graph of GFAP expression and statistical results in mice of all groups. The protein level of GFAP in the STZ group was significantly higher than that in the control mice (^∗^*p* < 0.05), but the expression could be inhibited by QTP (^#^*p* < 0.05). (b) Representative bar graph of CD11b expression and statistical results in the mice of all groups. The protein level of CD11b in the STZ group was significantly higher than that in the control mice (^∗^*p* < 0.05), but the expression could be inhibited by QTP (^#^*p* < 0.05). All data were expressed as means ± SEM. ^∗^*p* < 0.05 vs. Cont; ^#^*p* < 0.05 vs. STZ, *n* = 5.

Next, we postulated that glial cell activation played an important role in the treatment of QTP on these DM mice. To test the hypothesis, western blots were performed with antibodies of the astrocyte and microglial activation marker proteins GFAP and CD11b. As shown in Figure 2(a), STZ induced an obvious increase in protein expression level of GFAP in DM mice (*p* = 0.041), which could be prevented by the cotreatment of QTP (*p* = 0.036). DM mice also showed significant upregulated CD11b protein level after STZ treatment (*p* = 0.029), but the protein increase was prevented by the QTP too (*p* = 0.039) (Figure 2(b)). The present data here suggested that QTP might improve the executive function deficits in DM mice by inhibiting both astrocyte and microglial activation.

### 3.3. QTP Decreased the Level of TNF-*α* and MCP-1 in the Hippocampal Tissues of DM Mice

**Figure 3:**
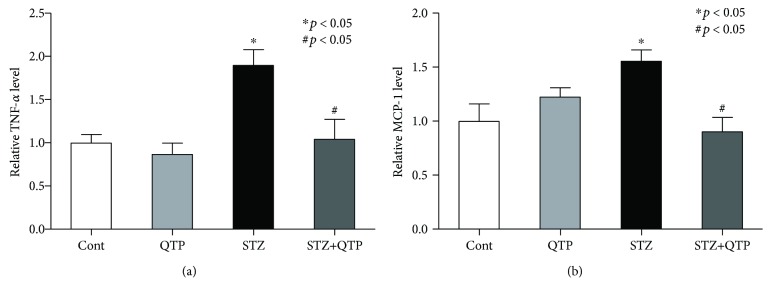
QTP decreased TNF-*α* and MCP-1 production in brain hippocampal tissues of DM mice. (a) TNF-*α* was measured with the ELISA kit in the brain hippocampal tissues in the mice of all groups. The protein level of TNF-*α* in the STZ group was significantly higher than that in the control mice (^∗^*p* < 0.05), but the expression could be inhibited by QTP (^#^*p* < 0.05). (b) MCP-1 was measured with the ELISA kit in the brain hippocampal tissues in the mice of all groups. The protein level of MCP-1 in the STZ group was significantly higher than that in the control mice (^∗^*p* < 0.05), but the expression could be inhibited by QTP (^#^*p* < 0.05). All data were expressed as means ± SEM. ^∗^*p* < 0.05 vs. Cont; ^#^*p* < 0.05 vs. STZ, *n* = 5.

**Figure 4:**
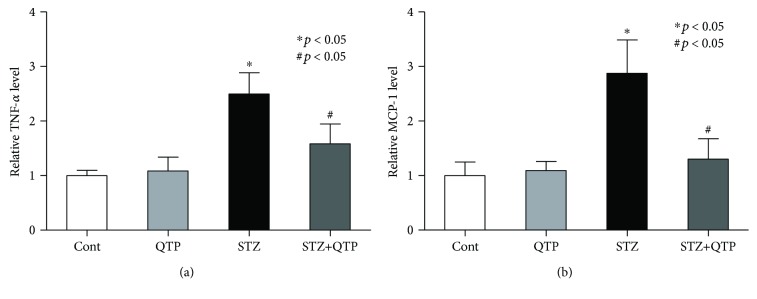
QTP decreased the release of TNF-*α* and MCP-1 kit in the brain CSF of DM mice. (a) TNF-*α* was measured with the ELISA kit in the brain CSF in the mice of all groups. The protein level of TNF-*α* in the STZ group was significantly higher than that in the control mice (^∗^*p* < 0.05), but the expression could be inhibited by QTP (^#^*p* < 0.05). (b) MCP-1 was measured with the ELISA kit in the brain CSF in the mice of all groups. The protein level of MCP-1 in the STZ group was significantly higher than that in the control mice (^∗^*p* < 0.05), but the expression could be inhibited by QTP (^#^*p* < 0.05). All data were expressed as means ± SEM. ^∗^*p* < 0.05 vs. Cont; ^#^*p* < 0.05 vs. STZ, *n* = 4.

In CNS, glial cells, astrocytes, and microglia are major players responsible for producing multiple cytokines [11]. Based on this, it is imperative to ask whether QTP could potentially regulate the cytokine expression profile in the brain tissue of the DM mouse. Initially, the expression level of TNF-*α* in hippocampal tissue was investigated with an ELISA kit. We found that QTP could reduce the upregulated level of TNF-*α* in the hippocampus (*p* = 0.012) (Figure 3(a)). The expression of another cytokine was explored in the following study. As shown in Figure 3(b), we found that the MCP-1 level was notably increased in the hippocampal tissue of the DM mouse (*p* = 0.028), which could be prevented by the QTP treatment too (*p* = 0.011) (Figure 3(b)). To study the released TNF-*α* and MCP-1 in the circulating system of CNS, we used the ELISA kit to assess the level of these two factors in CSF of the mice in all groups. We found that both of these two cytokines were significantly increased in CSF, but QTP could decrease the increased level effectively (Figure 4). These above results implied that QTP inhibited the activation of glial cells in CNS of DM mice and reduced the cytokine products from the glial cells.

### 3.4. QTP Prevented the Protein Loss of SYP and MBP in the Brain Hippocampal Tissues of DM Mice

**Figure 5:**
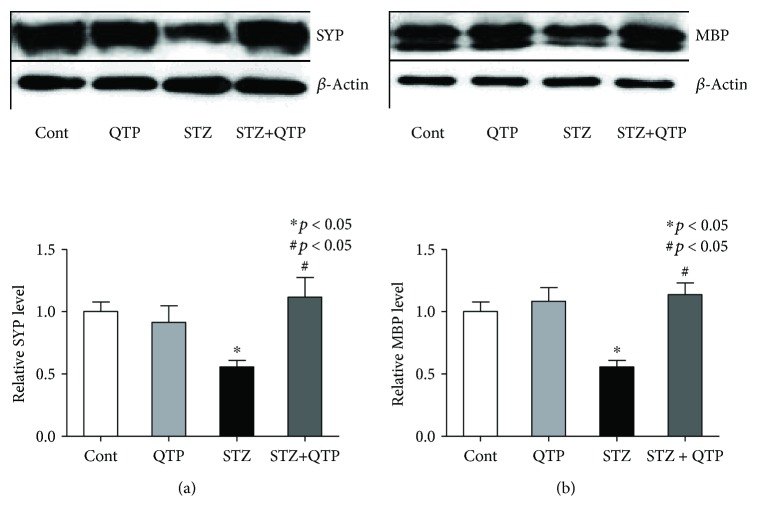
QTP prevented the protein loss of SYP and MBP in the brain hippocampal tissues of DM mice. (a) Representative bar graph of SYP expression and statistical results in the mice of all groups. The protein level of SYP in the STZ group was significantly lower than that in the control mice (^∗^*p* < 0.05), but the expression could be increased by QTP (^#^*p* < 0.05). (b) Representative bar graph of MBP expression and statistical results in the mice of all groups. The protein level of MBP in the STZ group was significantly lower than that in the control mice (^∗^*p* < 0.05), but the expression could be increased by QTP (^#^*p* < 0.05). All data were expressed as means ± SEM. ^∗^*p* < 0.05 vs. Cont; ^#^*p* < 0.05 vs. STZ, *n* = 5.

Memory and cognition are closely relevant with not only synaptic proteins, including synaptophysin (SYP) [12, 13], but also myelin-related proteins, such as myelin basic protein (MBP) [14, 15]. Here, we asked whether there was synaptic and myelin protein loss in the brains of mice exposed to STZ. Western blot assays were performed to evaluate the expression level of these two proteins. Our results demonstrated that STZ caused significant protein expression reduction of SYP and MBP in the brains of DM mice (Figure 5). When cotreated with QTP, the protein losses were notably reversed in these DM mice (Figure 5**)**.

## 4. Discussion

DM is a systemic disease that can adversely affect several organs of the body. Recently, effort has been made to explore the effect of DM on the brain since DM has been implicated in the development of neurological comorbidities [16]. DM often results in a multiple of complications in the brain including memory and cognitive decline. However, the exact pathophysiology of cognitive dysfunction in DM remains unclear so far. Therefore, no effective therapeutic strategies are available clinically.

Pathological changes in the hippocampus may contribute to the brain symptom of DM-associated complications by failing to maintain fair learning and memory function. Further characterization of changes in CNS of DM patients may help the development of new pharmacological interventions that are able to reverse the injury effects of diabetes on the brain and behaviors.

It has been well established in literature that neuronal dysfunction is closely associated with the increased expression of proinflammatory cytokines [17]. Inflammation in CNS normally is a protective response that helps the healing process; however, prolonged inflammation can lead to tissue damage [18]. In the present study, we established a DM mouse model by injecting STZ and measured the expression level of TNF-*α* and MCP-1 in these DM mice. As expected, the expression of these two cytokines was boosted in DM mice (Figures 3 and 4). These results were consistent with a previous report and our hypothesis. More importantly, we found that the increased cytokine expression could be reduced with cotreated QTP and the effect was associated with the improved memory, cognitive, and executive functions (Figure 1). In the present study, while focusing on the memory and cognitive function, we employed the puzzle box test to explore the abnormality of executive function as well. The puzzle box test is a problem-solving test in nature. Mice need complete escape tasks (puzzles) with increasing difficulty within 5 minutes in 3 consecutive days. The problem-solving task (puzzle box) reflects executive function in mice by showing the capability to figure out and solve the task (puzzle) [8]. To perform behaviors of executive function, mice are required to translate a goal-directed intention into motor behavior of increasing complexity. The beneficial effects of QTP on mice in the puzzle box test may not be due to the changes of anxiety-like behavior in mice in different groups, since there was no significant discrepancy in the latency to reach the goal zone among the groups in the baseline of problem solving (Figure 1). The importance of this finding is further highlighted by the key role of TNF-*α* in working memory formation of mice [19]. And MCP-1 also is a key contributor of cognitive deterioration [20]. Synaptic protein loss is a well-established molecular feature of memory and cognitive deficits in animals [21]. Consistently, we found that these DM mice showed significantly reduced expression of SYP. SYP is a protein in synaptic vesicles of the presynaptic membrane, and its accumulations in CNS white matter could serve as an immunohistochemical marker of axonal damage in myelin loss and neuroinflammation [22]. This led us to consider that SYP and the myelin protein MBP could be regulated by the STZ, which induced neuroinflammatory response and the subsequent neuronal damage in DM mice. MBP protein loss is closely associated with cognitive impairment in animal models [23]. While the expression level of both SYP and MBP was compromised in DM mice in the present study, QTP treatment effectively attenuated the protein loss (Figure 5).

Recent studies demonstrate that DM is a very important risk factor for Alzheimer disease (AD) [24]. Recent evidence implicates that insulin resistance contributes to the pathophysiology and clinical symptoms in AD [25]. Therefore, it is imperative to explore possible effective treatment strategies for the cognitive or executive dysfunction in the diabetic, even prediabetic, stage, which may prevent a significant amount of elderly patients to develop to AD. So far, there is no specific treatment available for these disorders in DM patients. QTP has been widely used clinically and showed a relatively better and safer profile in elderly patients because of its decreased propensity to cause the side effect of extrapyramidal symptoms [26]. In a recent study, QTP was also found to exert anxiolytic-like effects in old mice [7]; these additional beneficial effects may further support the application of QTP in elderly patients. In a nutshell, by finding the beneficial effect of QTP on the behavioral performance and axonal-related protein expression of SYP and MBP, our finding may provide new direction for the application of QTP. Meanwhile, our result may also provide a new possible option for the treatment of DM patients with mild cognitive impairment. There were limitations in the present study. As with most of antipsychotics, QTP has the potential side effect of metabolic status and may cause the fluctuation of blood glucose. Therefore, the dose and the treatment duration need be elaborated with further studies. Also, we administrated the QTP in drink water on a daily basis instead of injection for the reason of reducing the stressful impact on the mice. Even with the apparent advantage of this treatment method, the final dosage of each individual mouse exposed to QTP may be influenced by many factors. Therefore, other treatment methods, such as intraperitoneal injection, need to be used for the future extensive studies. Last, the open field in the puzzle box apparatus may influence the mood status of mice, which caused anxiety-like behavior during the 3-day test. QTP has been found to attenuate anxiety-like behavior in mouse models as well [7]. Therefore, the score in each task may be affected by the effect of QTP on the anxiety-like behavior instead of memory and executive functions in mice. To exclude this possibility, other memory and cognitive function tests which are less influenced by anxiety-like behavior, such as the water maze and the Y maze, should be included in the future studies.

## Data Availability

The data used to support the findings of this study are available from the corresponding author upon request.
